# Design and Test of a Biosensor-Based Multisensorial System: A Proof of Concept Study

**DOI:** 10.3390/s131216625

**Published:** 2013-12-04

**Authors:** Marco Santonico, Giorgio Pennazza, Simone Grasso, Arnaldo D'Amico, Mariano Bizzarri

**Affiliations:** 1 Center for Integrated Research—CIR, Unit of Electronics for Sensor Systems, Università Campus Bio-Medico di Roma, Via Alvaro del Portillo 21, Rome 00128, Italy; E-Mails: m.santonico@unicampus.it (M.S.); s.grasso@unicampus.it (S.G.); 2 Department of Electronic Engineering, University of Rome Tor Vergata; Via del Politecnico 1, Rome 00133, Italy; E-Mail: damico@eln.uniroma2.it; 3 Department of Experimental Medicine, Systems Biology Group, University La Sapienza, via Scarpa 14-16, Rome 00161, Italy; E-Mail: Mariano.Bizzarri@uniroma1.it

**Keywords:** chemical sensors, sensor array, electronic nose, electronic tongue, anthocyanins

## Abstract

Sensors are often organized in multidimensional systems or networks for particular applications. This is facilitated by the large improvements in the miniaturization process, power consumption reduction and data analysis techniques nowadays possible. Such sensors are frequently organized in multidimensional arrays oriented to the realization of artificial sensorial systems mimicking the mechanisms of human senses. Instruments that make use of these sensors are frequently employed in the fields of medicine and food science. Among them, the so-called electronic nose and tongue are becoming more and more popular. In this paper an innovative multisensorial system based on sensing materials of biological origin is illustrated. Anthocyanins are exploited here as chemical interactive materials for both quartz microbalance (QMB) transducers used as gas sensors and for electrodes used as liquid electrochemical sensors. The optical properties of anthocyanins are well established and widely used, but they have never been exploited as sensing materials for both gas and liquid sensors in non-optical applications. By using the same set of selected anthocyanins an integrated system has been realized, which includes a gas sensor array based on QMB and a sensor array for liquids made up of suitable Ion Sensitive Electrodes (ISEs). The arrays are also monitored from an optical point of view. This embedded system, is intended to mimic the working principles of the nose, tongue and eyes. We call this setup BIONOTE (for BIOsensor-based multisensorial system for mimicking NOse, Tongue and Eyes). The complete design, fabrication and calibration processes of the BIONOTE system are described herein, and a number of preliminary results are discussed. These results are relative to: (a) the characterization of the optical properties of the tested materials; (b) the performance of the whole system as gas sensor array with respect to ethanol, hexane and isopropyl alcohol detection (concentration range 0.1–7 ppm) and as a liquid sensor array (concentration range 73–98 μM).

## 1. Introduction

Sensors' potentialities can be greatly amplified by multidimensional arrangements in complex systems [1–3] or networks [4,5]. The organization of chemical sensors in arrays represents the basis for devices oriented to mimic the working principles of the human senses [6–8]. Among them olfaction and taste are the most reproduced by artificial versions [9–12] and both are widely exploited for medical applications [13–15] and in the monitoring of food production and preservation processes [16–19]. In spite of a fifteen-year history, electronic noses and tongues have not yet gained a satisfactory level of maturity as diagnostic or industrial technological tools for routine activities [20] even if the number of applications has been constantly increasing. Motivations for this gap can be found in a general lack of standardization among different results provided by the various technologies (transducers and sensing materials). Cross selectivity represents a problem when referred to the same sensing material with a sole transducer, but it has been solved by using the sensor arrays strategy combined with multivariate data analysis techniques [21]. This is the concept behind the electronic nose paradigm [22]. On the other hand, cross selectivity becomes an opportunity when referred to different transducers using the same material as sensing interface for the same sample in different phases (vapor and liquid). In this context the present work proposes to enlarge the concept of artificial olfaction and taste, designing an innovative multisensorial system based on the same bio-material as sensing element. Human beings contemporaneously use the five senses: senses contamination and interaction is called synesthesia [23]. The BIOsensor-based multisensorial system for mimicking Nose, Tongue and Eyes (BIONOTE) described in this paper represents the first step towards the realizing the strategy described above. In this case cross-sensitivity is more similar to ‘cross-sensoriality’, meaning the work made by different sensors to catch more complete information, mimicking synesthesia effects in senses mechanisms. In literature there are some papers recounting studies conducted in parallel with electronic noses and tongues [24,25], or combining gas sensors based on conductivity or mass changes and those based on optical properties [26] but they are exclusively based on data fusion, and data are provided by different experiments conducted with different devices. Among them it is worth mentioning that Rodríguez-Méndez *et al.* [24], prepared an excellent set-up for the integration of different systems (there named electronic panel), whose final result is a very interesting ‘chemical signature’ called multimodal feature vector. Cole *et al.* [27] reported in 2011 an interesting experiment of a simultaneous measurement of both a liquid and its vapor phase, with two transducers based on different sensing interfaces. In fact, while conducting polymers were used for vapor analysis, liquids were tested through their electrical properties, without a sensing material, because in this case physical parameters were measured instead of chemical ones. The simultaneous analysis of the vapor and liquid phase of the same sample, together with the test of its optical properties by mean of a single instrument based on the same sensing interface (the selected sensing material of biological origin), has similarity with an ‘artificial synesthesia’ that we want to call ‘multi-sensorial’ approach. This simultaneous approach for liquid-vapor measurement seems not to be useful in case of gas samples, but looking at the medical applications of this system, it emerges as a novel source of information. Many medical studies are devoted to body fluids more involved in the diseases under test, while a multidimensional approach suggests the analysis of the whole set of information revealed by human metabolism [13].

Anthocyanins are used as BIONOTE sensing materials. Anthocyanins are natural pigments widely distributed in nature. They are produced by plants as secondary metabolites responsible for the pigmentation of many flowers, fruits and vegetables [28]. Depending on the plant source, a great amount of anthocyanins different in type, concentration and characteristics have been described [29]. Regardless to the heterogeneous nature of the molecule, all the anthocyanins belong to the flavonoid group of polyphenols and share a common structure. The main part of an anthocyanin is the flavylium cation, which contains conjugated double bonds and is responsible for light absorption. This peculiar feature makes the pigments to appear colored to the human eye [30]. The most important aglycone forms of anthocyanins, also called anthocyanidins, are pelargonidin, cyanidin, peonidin, delphinidin, malvidin, and petunidin. Anthocyanidins as such are highly unstable molecules, therefore these are mainly found in nature in a variety of modified forms [31]. The attachment of glycosyl units and acyl groups as well as the site of their bonding affects both the stability and reactivity of the anthocyanin molecule significantly [32,33]. Besides their compelling role as potent antioxidant agents [34], anthocyanins have raised a great interest as natural dyes. The ionic nature of anthocyanins enables the changes of the molecule chemical structure according to the prevailing pH, resulting in different colors and hues at different pH values [35,36]. The red flavylium cation is the predominant form in acidic aqueous solutions, while the violet and blue species dominate in alkali media. The color of an alkali solution can be reverted to the color of an acidic media by shifting the pH back to lower values. However once anthocyanins undergo pyrylium ring opening and the ionic chalcones have been formed, reversibility of the pigmentation cannot be achieved any more [37]. In addition to pH, anthocyanins' stability is affected by other parameters such as solvent nature, temperature, light and oxygen exposure [38]. *In vivo* the stabilization of the colored structure of the anthocyanin molecule occurs via different mechanisms involving intra- and inter-molecular interactions. Thanks to the complexity of the biological matrix in which the molecules are naturally embedded, the anthocyanins can interact with themselves as well as with other compounds including co-pigments and metal ions [39]. So far, the anthocyanin class of molecules with its extensive complexity and heterogeneous chemical behavior seems to represent an excellent candidate for fundamental studies in the material science context and for a variety of innovative applications, by using them as sensing materials for opportune transducers.

In this work, Quartz Micro Balances (QMBs) are the transducers used for the gas sensor array. QMBs have a very high resolution power (round 0.7 ng for a 20 MHz resonant crystal in thickness shear mode), and, as mass sensors, they are intrinsically non selective. The liquid sensor array is composed of screen-printed gold electrodes (Aux.: Pt; Ref.: Ag). As far as we know anthocyanins have never been used as sensing material (with the exception of some applications as optical sensors).

## 2. Materials and Methods

### 2.1. Anthocyanin Extraction and Quantification

A red rose (*Rosa europeana*), a blue hortensia flower (*Hydrangea macrophylla*) and a red cabbage (*Brassica oleracea* var. capitata f. rubra) were used as source of the natural pigments investigated in the present study. For extraction, fresh plant tissues (10 g) were harvested and immediately incubated for 2 h at room temperature in an acidic methanolic solution (0.1% HCl v/v). Crude extracts were clarified by centrifugation at 10.000 rcf and 4 °C for 15 min to remove any traces of particulates. The recovered supernatant was then stored at −20 °C until use. Total phenolic content of purified extracts was spectrophotometrically determined by the Folin-Ciocalteu method [40] and results were expressed as gallic acid equivalent (GAE) in g/100 g of material. The concentration of polyphenols in samples was derived from a standard curve of gallic acid (Sigma-Aldrich, Milan, Italy) ranging from 1 to 7.5 μg/mL (Pearson's correlation coefficient: r^2^ = 0.99). The total anthocyanin concentration was estimated by the pH differential method [41]. The absorbance values were related to anthocyanin content using the molar extinction coefficient calculated for cyanidin-3-O-glucoside.

### 2.2. Absorbance Spectra Determination

Optical properties of anthocyanins extracts were investigated recording the absorbance in the ultraviolet-visible (UV-Vis) spectral region ranging from 230 nm to 800 nm. To evaluate the color transition four different buffer mixtures were prepared: solution A (25 mM potassium chloride, pH 1.0), solution B (0.4 M sodium acetate, pH 4.5), solution C (0.4 M sodium acetate, pH 6), solution D (0.1 M disodium hydrogen phosphate, pH 7.5). Samples were diluted in the proper solution before the analysis so as not to exceed the buffer capacity of the reagents. Once analyzed each sample was recovered, placed in polypropylene vials and challenged for different storage conditions. Anthocyanins extracts diluted in aqueous buffered solutions were kept independently for 14 days at room temperature and 4 °C or for 2 h at 40 °C. All the spectrophotometric analyses were performed on a Shimadzu UV-1800 instrument (Shimadzu, Milan, Italy).

### 2.3. HPLC UV/Vis Analysis

Anthocyanins composition of the extracts was determined on a Shimadzu Prominence HPLC-20AD XR system equipped with a SPD-20 AD UV detector. A sample (20 μL) of the acidic methanol extract was injected onto an analytical reverse phase C18 Discovery HS column (250 × 2.1 mm, 5 μm particle size; Supelco/Sigma-Aldrich, Bellefonte, PA, USA) and the separation was carried out eluting with a high-pressure binary gradient. The mobile phase was composed of 5% formic acid in water (Solvent A) and methanol (Solvent B). The gradient conditions were as follows: from 10% to 20% B in 30 min, from 20% to 25% B in 5 min, from 25% to 60% B in 20 min, from 60% to 100% B in 1 min, then held for 10 min before returning to the initial conditions. Chromatograms were recorded at absorbance of 520 nm and 280 nm. Data acquisition and processing were performed with the Shimadzu LC Solution software. Three separate analyses were carried out for each sample.

### 2.4. Transducers

The transducers used for the gas sensor array are quartz crystals with a resonance frequency of 20 MHz in the thickness shear mode (fabricated by CNR IMM, Rome, Italy) [42]. The sensing material covering each crystal was deposited by drop casting and was a combination of anthocyanins extracted from three different plant tissues: red rose, red cabbage, blue hortensia. The list of the six sensors is: sensor 1, RR (red rose extract 65 mM); sensor 2, BH (blue hortensia extract 65 mM); sensor, 3 RC (red cabbage extract 65 mM); sensor 4, RRS (red rose extract 65 mM + sucrose 10 mM); sensor 5, RCS (red cabbage extract 65 mM + sucrose 10 mM); sensor 6, BHS (blue hortensia extract 65 mM + sucrose 10 mM). To test system reproducibility, each sensor was prepared in triplicate depositing 10 μL of the specific sensing material and then letting it air dry.

The liquid sensor array is composed of screen-printed gold electrodes (Aux.: Pt; Ref.: Ag) fabricated by DropSens S.L. (Llanera (Asturias), Spain); the working (4 mm diameter) electrode is made of gold, the counter electrode is made of platinum, the reference electrode is made of Ag and electric contacts are made of silver. Its dimensions (3.4 × 1.0 × 0.05 cm—Length × Width × Height) are ideal for working with 50 μL volume. The sensing material used to functionalize the electrode surface was a combination of anthocyanins extracted from red cabbage and polymerized with 4.5% (w/v) agarose. Liquid sensors were freshly prepared before each analysis by casting 10 μL of the sensing material solution onto the working electrode surface and then letting it air drying.

**Figure 1.**
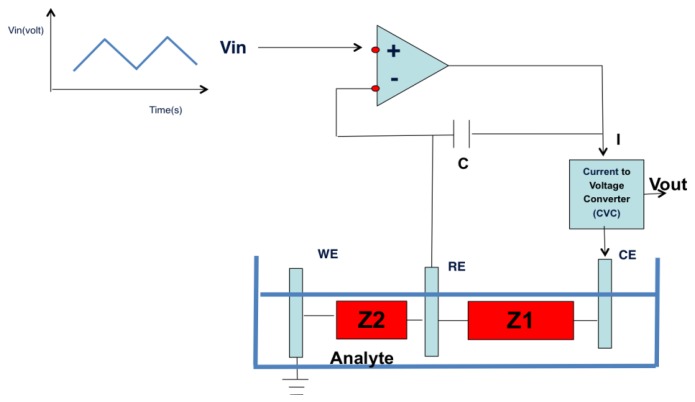
Electronic interface for liquid sensor system. The input signal is a triangular function. The output signal is measured by the working electrode and transduced in an output current by an opportune converter.

The electronic interface for voltage input and for the reading of the out current is shown in Figure 1. Opamp 1 allows the reference electrode to keep a selected voltage (also time dependent) without having current through it. Opamp 2 transforms the working electrode current in an output voltage representing the overall circuit response. The electronic board controlling the whole system is based on a STM 32 F303VC, fabricated by ST Microelectronics (Geneva, Switzerland).

### 2.5. Calibration Set-Up

In order to perform gas and vapor measurements in the concentration range below 1 ppm, a strategy based on permeation tubes has been utilized. A similar setup was already used with the electronic nose of the University of Tor Vergata and it is fully described in a work by the same authors of the present paper [43]. The selected compounds were ethanol, hexane and isopropyl alcohol. The concentration levels tested in these experiments for ethanol were: 0.5, 1.5, 3.2, 4.5 and 6.1 ppm (five measurements each). The concentration levels tested in these experiments for hexane were: 0.7, 2.1, 4.1, 6.3 and 7.2 ppm (five measurements each). The concentration levels tested in these experiments for isopropyl alcohol were: 0.6, 1.8, 3.9, 5.4 and 6.3 ppm (five measurements each). For the test of the liquid sensor array cyclic voltammetry in the range from −1 to 1 Volt has been employed using a triangular function at 1 mHz and at 10 mHz. Liquid samples were enclosed in 3 mL polyethylene vials normally used for photospectrometry.

### 2.6. Data Analysis

The parameters (calculated for each measured compound) provide the elements needed to assess the performance of the sensors, and they could be used to fill-in a sort of data sheet of the instrument. These technical notes could orient the device for specific applications. This information can be also used for the system re-calibration and standardization using one of the tabled compounds, depending on the specific application. The sensors' behavior has been analyzed taking into account the following different aspects:

- calibration curve (QMB frequency shift versus VOC concentration)
- sensitivity (slope of the linearized calibration curve)
- experimental Limit of Detection (LOD) determined around the left end part of the calibration curve
- theoretical LOD (estimated considering a signal to noise ratio S/N equal to 1)

Sensitivity and LOD role consists of supporting the e-nose pertinence to many different applications.

For the multivariate data analysis approach, Principal Component Analysis (PCA) has been used as an unsupervised method; as a supervised method Partial Least Square Discriminant Analysis (PLS-DA) has been applied, with Leave-One-Out as Cross-validation criterion. PCA and PLS-DA have been performed using the PLS-Toolbox SW (Eigenvector, Wenatchee, WA, USA) in the Matlab environment ( The Mathworks, Natick, MA, USA).

## 3. Results

### 3.1. Gas Sensor Array

**Figure 2.**
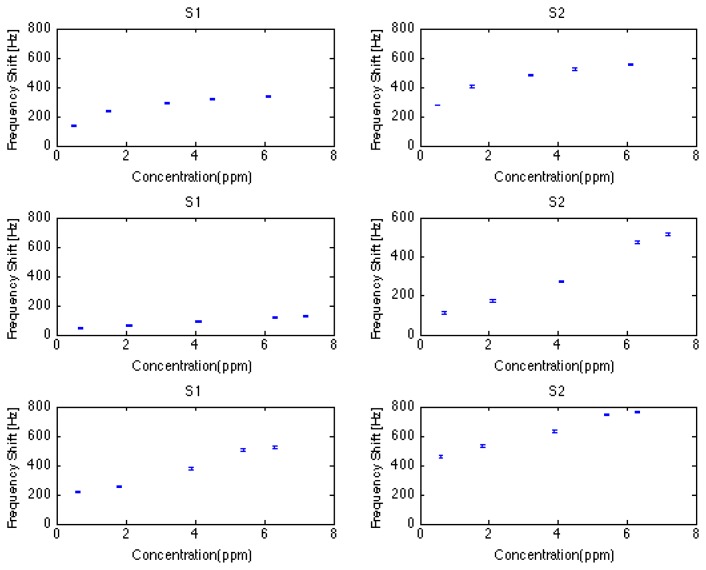
Calibration curves of S1 and S2 (QMB sensors) for three compounds: ethanol, hexane and isopropanol.

Calibration curves of the two best-performing sensors (S1 and S2) are reported in Figure 2.

### 3.2. Response to Ethanol

Reproducibility can be observed by the error bars and the fact that discrimination among the measured concentration levels is not disturbed by overlapping standard deviation, being it smaller than the difference calculated between contiguous concentration levels.

Calibration curves are not linear and they can be fitted *prima facie* by a Langmuir Isotherm curve. Sensor 3 does not show a good performance, being its sensitivity very low and its calibration curve very similar to a saturation behavior. Sensitivity goes from a minimum value of about 9 Hz/ppm to a maximum value of about 93 Hz/ppm.

The experimentally measured LOD is 0.5 ppm, with a S/N ratio still far from 1. In fact, this is the lowest measured level of ethanol concentration, and the relative response values range from a minimum of 19 Hz to a maximum of 278 Hz. Given the noise level of 3 Hz, the theoretical LOD goes from 333 ppb to 32 ppb.

### 3.3. Response to Hexane

Reproducibility can be observed by the error bars as in the previous case (ethanol). Calibration curves appear to be linear. Further, in this case, three different behaviors can be observed: sensor 3 does not show a good performance, being its sensitivity very low and its calibration curve similar to a saturation behavior; sensors 1, 2 and 6 show a rather good sensitivity: from a minimum value of about 17 Hz/ppm to a maximum value of about 72 Hz/ppm; sensors 4 and 5 show a good sensitivity: from a minimum value of about 5.5 Hz/ppm to a maximum value of about 7.7 Hz/ppm.

The experimentally measured LOD is 0.7 ppm, with an S/N ratio still far from 1. In fact, this is the lowest measured level of hexane concentration, and the relative response values range from a minimum of 26 Hz to a maximum of 112 Hz. Given the noise level of 3 Hz, the theoretical LOD goes from 540 ppb to 42 ppb.

### 3.4. Response to Isopropyl Alcohol

Reproducibility can be observed by the error bars as before (ethanol and hexane). Calibration curves appear to be linear. Furthermore, in this case, three different behaviors can be observed: sensor 3 does not show a good performance, being its sensitivity low and its calibration curve similar to a saturation behavior; sensitivity goes from a minimum value of about 10 Hz/ppm to a maximum value of about 110 Hz/ppm. The experimentally measured LOD is 0.6 ppm, with an S/N ratio, still far from 1. In fact, this is the lowest measured level of isopropyl aclohol concentration, and the relative response values range from a minimum of 32 Hz to a maximum of 454 Hz. Given the noise level of 3 Hz, the theoretical LOD goes from 300 ppb to 27 ppb.

### 3.5. Liquid Sensor Array

**Figure 3.**
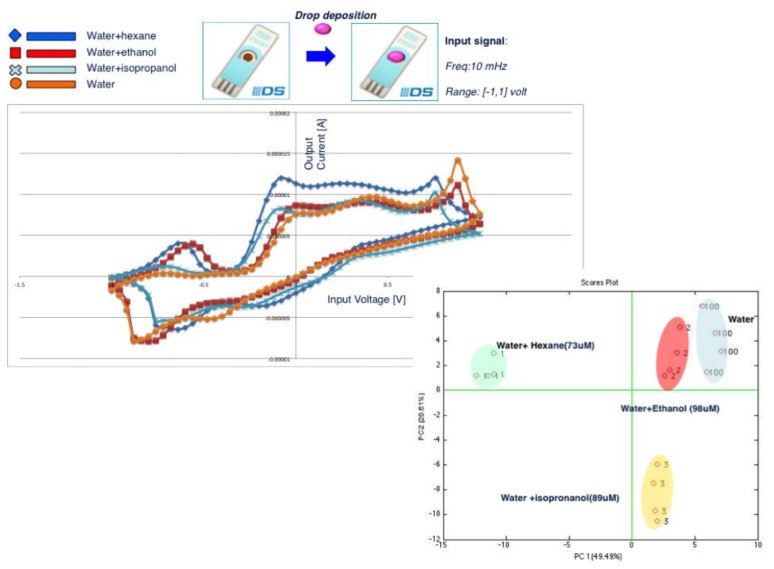
The voltage modulation applied to the liquid sensor provides in each time a value of current. The peaks of current are referred to different redox processes. All the current responses represent an electrochemical pattern of the sample (each marker corresponds to a different specimen). The scores plot obtained from a PCA model built on the collected data shows different clusters for different compounds.

The same compounds tested with gas sensors have been measured in the liquid phase: ethanol, hexane and isopropyl alcohol diluted in water at 98 μM, 73 μM and 89 μM, respectively. Figure 3 shows the obtained results. In Figure 3, voltammetry cycles show different curve shapes, evidencing current peaks for each compound, which appears to be different in terms of voltage values (horizontal axis) at which the peaks occur and in terms of the intensity (vertical axis) of the peaks themselves. Each curve has been treated as a single measure composed by 100 input voltage values and the 100 corresponding output current values. The array of the 12 (raw) measurements (four for each compound) and of the 100 columns has been analyzed by Principal Component Analysis (PCA). The scores plot of the first two Principal Components (PCs), accounting for 77.5 of the explained variance, is reported in Figure 3. Four distinct clusters can be observed for the four compounds: water and the three water-based solutions. Polar and non polar compounds are distinguished along the Principal Component 1 (PC1), while along the PC2 it is possible to discriminate the three polar compounds. The dimension of the liquid sensor patterns can be increased by mean of three strategies: (a) using the same electrode with the same sensing material at different frequencies of the input voltage function; (b) using different electrodes with same sensing materials; (c) using the same electrodes with different sensing materials. The set-up used for the measurements reported in this work is: Pt electrodes functionalized with the red cabbage anthocyanins extract and with an input signal frequency of 10 mHz.

### 3.6. Optical Properties

Plant materials from red cabbage, rose and hortensia flowers were incubated in an acidified alcoholic mixture at room temperature and in the dark to extract anthocyanins without disrupting the general architecture of the tissues. At the end of the incubation, the petals of the flowers and the leaves of the vegetable matter were completely white, whereas the recovered extraction solutions appeared intensely colored.

**Figure 4.**
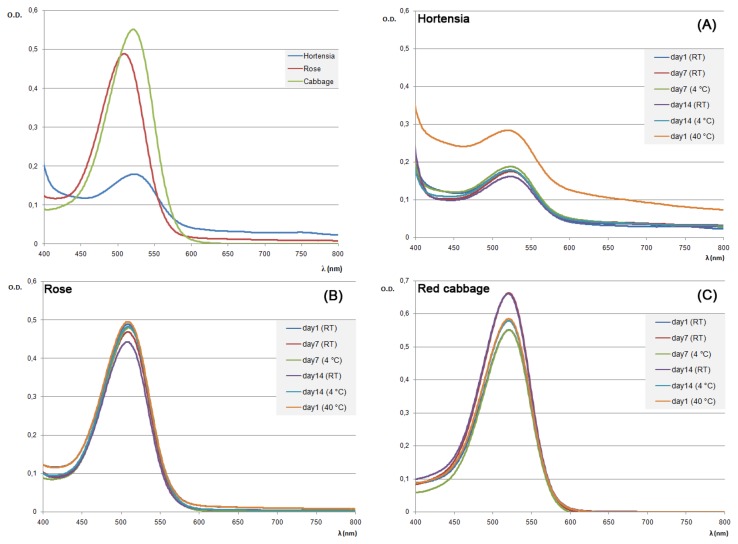
Optical spectra of different sensing materials in different conditions. The wavelength at which the anthocyanin chromophore shows the maximum absorbance in the pH 1.0 solution is 520 nm. The optical stability of sensing material (hortensia panel (**A**), rose panel (**B**), cabbage panel (**C**)) has been monitored for a period of 14 days at different temperatures (room temperature (RT), 4 °C and 40 °C).

Each extract has been subsequently characterized by three physico-chemical parameters: the total polyphenolic content, the total monomeric anthocyanin content and the stability under different storage conditions. According to the well-known antioxidant properties of anthocyanins, the Folin-Ciocalteu assay was applied as preliminary analysis to determine the total phenolic content. The highest values were observed in the rose extract with 5.75 g/L (GAE). The amount of phenolic compounds measured in the red cabbage and the hortensia flower extracts was almost the same, 1.46 g/L and 1.68 g/L (GAE) respectively, with values significantly lower than the rose one. As a major drawback of the general method used for preparing anthocyanin-rich extracts from plant materials, all the molecules with polar character and phenolic groups were co-purified and quantified by the Folin-Ciocalteu reagent. To obtain a better estimation of the amount of anthocyanin pigments within the extracts, the pH differential method was performed. Samples were diluted with aqueous pH 1.0 and 4.5 buffers and absorbance measurements were taken at 520 nm, the wavelength at which the anthocyanin chromophore had the maximum absorbance in the pH 1.0 solution (Figure 4).

**Figure 5.**
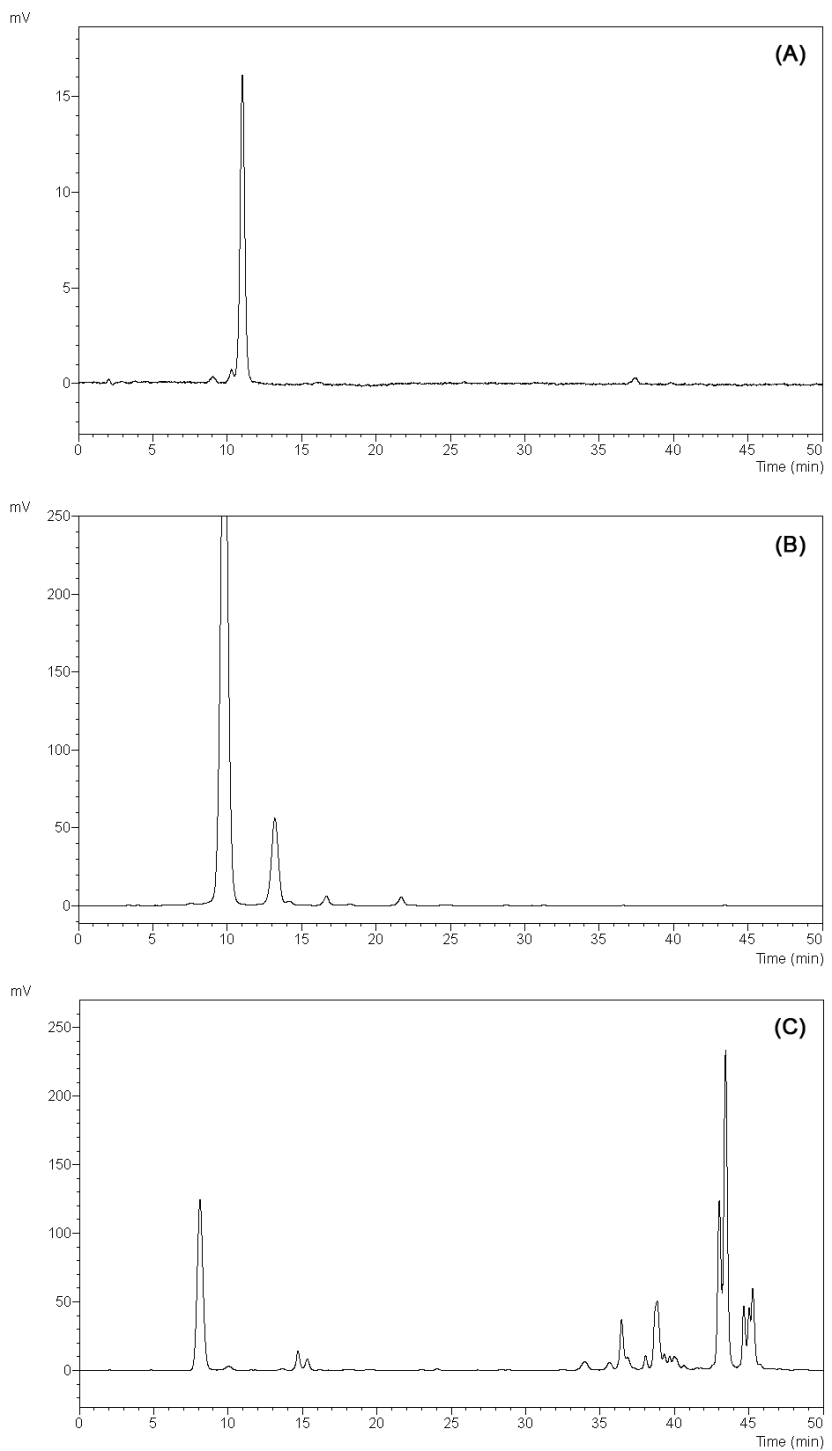
HPLC-UV/Vis chromatograms acquired at 520 nm of (**A**) blue hortensia, (**B**) red rose and (**C**) red cabbage anthocyanins extracts.

Turbidity has been also taken into account by measuring the absorbance at 700 nm and subtracting this from the values recorded at the wavelength of maximum adsorption. In contrast with the previously reported results, the red cabbage extract showed the highest concentration of anthocyanins with values comparable to rose extract. On the other hand, the amount of anthocyanin pigments measured in the hortensia extract was significantly lower than expected. To investigate further the observed discrepancy, anthocyanins in each extract were characterized by performing HPLC analysis (Figure 5).

Identification of the anthocyanins was attempted by comparison of the retention times of the observed peaks with those of standard molecules and well-known anthocyanin-rich matrices. Results obtained from analytical separation confirmed a general lower complexity of the latter in terms of total amount as well as number of different anthocyanin molecules (Figure 5). In the hortensia chromatogram four peaks were detected, with only one peak being the absolutely dominant component (Figure 5A). Conversely, rose and cabbage chromatograms showed a more complex anthocyanins composition. In particular, the rose chromatogram highlighted the presence of at least six different anthocyanins with two of them representing 92% of the total amount of molecules (Figure 5B, peaks 1 and 2), whereas the red cabbage extract showed an even more complex and heterogeneous anthocyanin composition (Figure 5C). Additionally, when the samples were analyzed by HPLC and detected by UV absorption a manifold presence of co-pigments in all the extracts was highlighted (data not shown). Exploiting further the good correlation between chemical characteristics and colorimetric information, the anthocyanin extracts were exposed to different storage conditions and their structural stability evaluated by measuring the absorbance behavior along an UV-Vis spectrum (Figure 4b). At very acidic pH (pH 1.0) the anthocyanins rich extracts resulted stable along a period of 14 days in all the storage conditions tested (room temperature, 4 °C; Figure 4). Nevertheless, anthocyanins stability in slightly acidic (pH 4.5 and pH 6.0) and alkaline (pH 7.5) environments was affected in an extract specific fashion. The anthocyanins deriving from cabbage were protected from degradation even after 14 days whereas the rose and hortensia extracts resulted unstable, especially in alkaline conditions (data not shown). Hortensia extract loss the distinctive absorption peak after one day of storage while rose anthocyanins resulted more stable undergoing degradation around at 7 days. In both cases, the degradation was observed with the same occurrence modality independently from the storage condition. Furthermore, incubating the three extracts for 2 h at 40 °C did not alter the absorbance spectra in any of the pH (from pH 1.0 to pH 7.5) tested (data not shown).

### 3.7. Gas-Liquid Sensor Synergy

**Figure 6.**
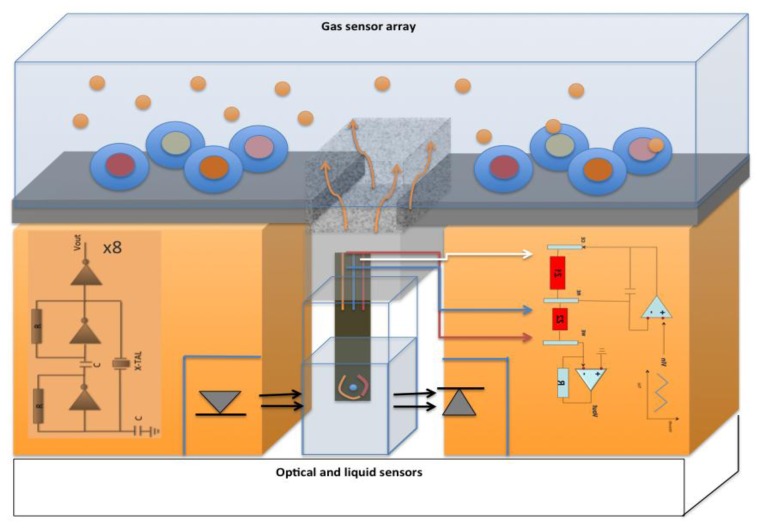
Concept design of a system composed of three parts: optical transduction, liquid sensors and gas sensors array. The sample under test could be contemporaneously analyzed by the three techniques.

**Figure 7.**
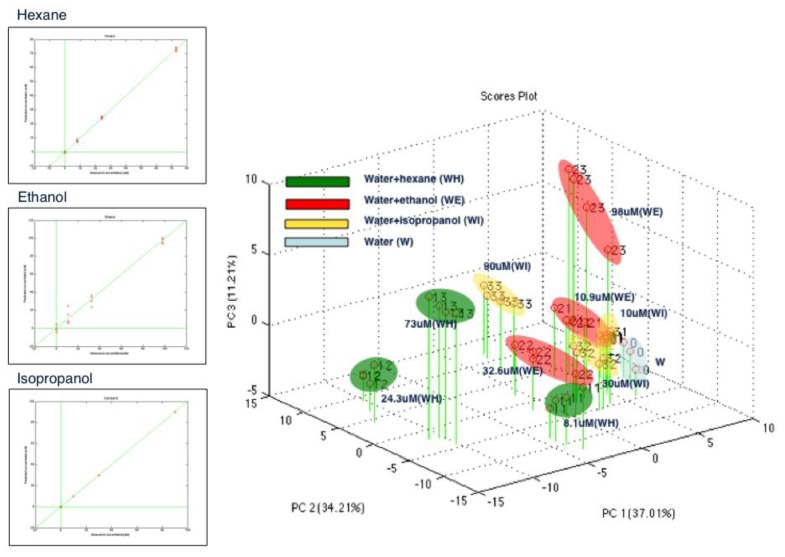
The liquid sensors have been calibrated to different solutions of water with addition of hexane, ethanol, isopropanol (**Left**). The score plot obtained by the principal component analysis model shows a discrimination of the mixtures (**Right**).

Using the setup reported in Figure 6, the water-based solutions analyzed by the liquid sensors (see the section on gas sensor array calibration) have been also measured simultaneously with the liquid and the gas-vapor sensor arrays. This setup has been tested as a proof of concept. Each compound has been measured at three concentration levels: ethanol (10.9, 32.6, 98 μM); hexane (8.1, 24.3, 73 μM); isopropanol (10, 30, 89 μM). The multidimensional approach already described has been here used calculating a Partial Least Square Discriminant Analysis (PLS-DA) model on the entire data set array (90 measurements, 10 for each concentration level × 100 potentials, from −1 V to 1 V; steps of 20 mV). The calculated PLS-DA models show a rather high degree of efficiency in the concentration discrimination, as shown in Figure 7, left panel. The Root Mean Square Error in Cross Validation (Leave One Out criterion has been used), 0.066, 0.303, 0.182 μM for ethanol, hexane and isopropanol respectively, are promising and relatively low. The score plot of the first three Latent Variables (LVs) reported in the right panel of Figure 7 shows the ability of the system to sharply discriminate between different solutions at different concentration levels. Vapor released by each solution has been simultaneously measured, and the demonstration of the liquid-gas sensor system functionality is given by the fact that the three gas mixtures have been correctly discriminated. The identification of the different gas concentration levels is not possible in this configuration, because the vapor headspace rapidly reaches the saturation vapor pressure. Anyway this is not the purpose of the liquid-gas sensor synergy, which is instead oriented to the analysis of complex biological samples (urine, sputum, sweat, beverage) in order to acquire the overall information represented by each specimen. In Figure 6 the arrangement to be realized for the optical reading of the sample, exploiting the peculiarity of the anthocyanins is also preconized.

## 4. Conclusions

This paper presents a series of novelties, which are here listed in order to better evidence the scientific contributions of this study.

- Due to the optical properties of anthocyanins, the potentialities of the BIONOTE system can also be improved by an optical transduction mechanism.
- Anthocyanins have never been used as chemical sensing elements and the ones used here have been well characterized in terms of optical spectrum and HPLC analysis.
- The electronic apparatus proposed here for the liquid sensor array is proven to be stable with also a very low noise output voltage.
- The multivariate analysis technique used for the liquid sensors data provides a novel methodology to exploit voltammetry outcomes enlarging data dimensionalities via virtual sensor generation (different applied voltages, different electrodes, different sensing material).
- The future development of this research will look towards a number of new possibilities of applications in the field of medicine, of food industry and environmental monitoring. In these contexts BIONOTE, the instrument illustrated here, could perform a simultaneous chemical analysis of the liquid and vapor phase of a sample using the same anthocyanins as chemical interactive material.
